# Prevalence of pelvic floor dysfunction in recreational athletes: a cross-sectional survey

**DOI:** 10.1007/s00192-023-05548-8

**Published:** 2023-05-10

**Authors:** K. Gillian Campbell, Mark E. Batt, Avril Drummond

**Affiliations:** Faculty of Medicine and Health Sciences, Queens Medical Centre, University of Nottingham, Room B302, B Floor, Queens Medical Centre, Nottingham, NG7 2HA UK

**Keywords:** Female athlete, Pelvic floor, Incontinence, Pelvic organ prolapse, Prevalence

## Abstract

**Introduction and hypothesis:**

Pelvic floor dysfunction (PFD) affects many women and participation in elite sport and high-impact exercise has been reported as a potential risk. However, few studies have investigated the effects of exercising at recreational levels on PFD. Our aim was to investigate levels of PFD in women exercising at, or above, UK guidelines for health and compare them with levels in non-exercisers.

**Method:**

Data on levels of PFD and potential risk factors (age, hormonal status, body mass index, constipation, parity, forceps delivery, and recreational exercise) were collected using a cross-sectional survey distributed via social media. The International Consultation Incontinence Questionnaire (ICIQ) Urinary Incontinence Short Form was used to estimate prevalence of urinary incontinence (UI). Selected questions from the ICIQ vaginal symptom and bowel symptom questionnaires were used to estimate prevalence of anal incontinence (AI) and pelvic organ prolapse (POP). Logistic regression analysis was used to compare exercisers and non-exercisers after adjusting for potential confounders.

**Results:**

We recruited 1,598 adult women (1,141 exercisers and 457 non-exercisers). The majority were parous. High prevalence of UI (70%), AI (52%) and POP (18%) was reported. No significant association was found between recreational exercise and PFD despite adjustment for confounders, or further investigation regarding exercise involving impact, although some increased reporting of AI was seen in those exercising for over 10 hours per week.

**Conclusion:**

High levels of all PFD were reported but no significant association was found between recreational exercise and symptoms. However, data suggest that women modify their exercise regimes as required. Few symptomatic women sought professional help.

**Supplementary information:**

The online version contains supplementary material available at 10.1007/s00192-023-05548-8

## Introduction

Pelvic floor dysfunction (PFD), which includes urinary incontinence, anal incontinence and pelvic organ prolapse (POP) [1], causes embarrassment and distress, limits many aspects of life [2] and affects many women [3]. It is accepted that childbirth, obesity and aging are risk factors for PFD [4] but recent evidence suggests that the prevalence of urinary incontinence in young, nulliparous athletic women is 2.77 times higher than in their sedentary counterparts [5]. Other reports suggest that high-impact activities, e.g. cheer leading, may be linked with increased levels of anal incontinence (AI) [6]. However, regular participation in sport and exercise confers multiple health benefits [7, 8]. Current UK recommendations are that adults should exercise at moderate levels or above for a minimum of 150 min each week, over three sessions [9]. Urinary incontinence (UI) can be a barrier to exercise [10] and concern regarding potential risks to the pelvic floor, as reported in elite athletes, may cause health professionals and women to question the safety of engaging in sport and exercise, for fear of aggravating symptoms or increasing the risk of developing PFD. Although studies have investigated whether the risk of PFD is higher in elite athletes than in sedentary individuals [11] and in younger women [6, 12], only a few have reported levels within a broad range of recreational athletes [13, 14]. Therefore, the objectives of this study were:

1. To investigate the levels of PFD reported by women who exercise at, or above UK guidelines for healthy living and in those who are more sedentary.
2. To investigate any association between PFD and taking part in sport at a recreational level.

## Materials and methods

### Study design

This was a cross-sectional survey specifically designed to investigate levels of UI, AI and POP in a convenience sample of adult women, reported using Strengthening the Reporting of Observational Studies in Epidemiology guidelines [15]. The study steering group, comprising the authors and a PPI member, designed the survey and developed it on Jisc Online surveys. The survey contained 37 questions divided into sections so that participants could bypass questions that did not apply to them. It was initially piloted with 31 participants recruited from administrative and academic staff from the School of Health Sciences, University of Nottingham, and a local physiotherapy clinic, to identify any issues with the language or question format. Minor signposting problems and issues with terminology identified were resolved.

### Sample size

In order to investigate a predicted potential significant difference of 10% in prevalence of PFD between recreational exercisers [14] and the general female population [16], with significance level set to 0.05 and 80% power, we aimed to recruit a minimum of 800 participants: 500 exercisers and 300 non-exercisers.

### Participants and recruitment

Adult women were invited to take part via advertisements, which were widely distributed on social media networks (Facebook, Twitter, Instagram and LinkedIn) and using snowball methodology (asking people to share the information with others). Posters were also distributed to sports clubs, workplaces and physiotherapy clinics for display on websites and notice boards. QR codes linked directly to the survey. Advertisements highlighted that ALL women were invited to take part: both those who did and those who did not exercise and both those with and those without any pelvic floor symptoms. Data collection took place between 6 May and 31 July 2022.

### Outcome measures

To determine the prevalence of UI, AI and POP (as defined by the International Urogynaecological Association (IUGA)/International Continence Society (ICS) joint report) [1], we collected data using patient-reported outcome measures. We used the International Consultation Incontinence Questionnaire (ICIQ) Urinary Incontinence Short Form (ICIQ-UI-SF) [17] in its entirety, and specific questions of interest from ICIQ bowel symptom [18] and ISIQ vaginal symptom questionnaires [19]. Inclusion of all three questionnaires in full would have resulted in a prohibitively time-consuming survey, likely to deter participation.

Those who reported “never” in response to the question “How often do you leak urine?” were classified as continent of urine, and severity was defined by the ICIQ-UI-SF severity score [20]. UI was further subdivided into stress UI (SUI), urgency UI (UUI) and mixed UI (MUI) based on the answers to “When does urine leak?” Anal continence was identified in those who answered, “always” to the question “Are you able to control leakage of stool or flatus (wind) from your back passage?” Responding “never” to the question “Are you aware of a lump or bulge coming down in your vagina?” was taken to indicate the absence of POP. Awareness of a lump or bulge in the vagina has been associated with the presence of a grade 2 POP, although this may underestimate the true prevalence of this dysfunction [21]. Age, menopausal status, body mass index (BMI), constipation (defined as regularly having to strain to open bowels), parity and type of delivery were considered potential risk factors for PFD and possible confounders.

Recreational athletes were defined to be those who met and exceeded the UK Chief Medical Officer’s guidelines for healthy living of 150 min a week [9]. This was further subdivided into high-impact (sports involving both feet leaving the ground at the same time, e.g., running, high impact gym or trampolining) and low-impact (one foot always in contact with the ground or body weight supported, e.g. walking, cycling, kayaking) or both.

Additionally, participants were asked if they had sought professional help for PFD, and if they regularly performed pelvic floor exercises. The final open question gave participants an opportunity to record comments or additional information regarding previous answers.

### Statistical analysis

Statistical analysis was performed in SPSS statistical software version 28 (IBM, Chicago, USA). Demographic data were reported using frequencies with percentages or means with standard deviations (SD). Prevalence was reported as frequency and percentage, and Pearson’s Chi-squared test was used to investigate any differences in prevalence of PFD between non-exercisers and exercisers. Missing data were reported. Risk factors for PFD were estimated by logistic binomial regression analysis, reported as adjusted odds ratios (OR) with 95% confidence intervals (CI), with the significance level set to 0.05.

## Results

**Fig. 1:**
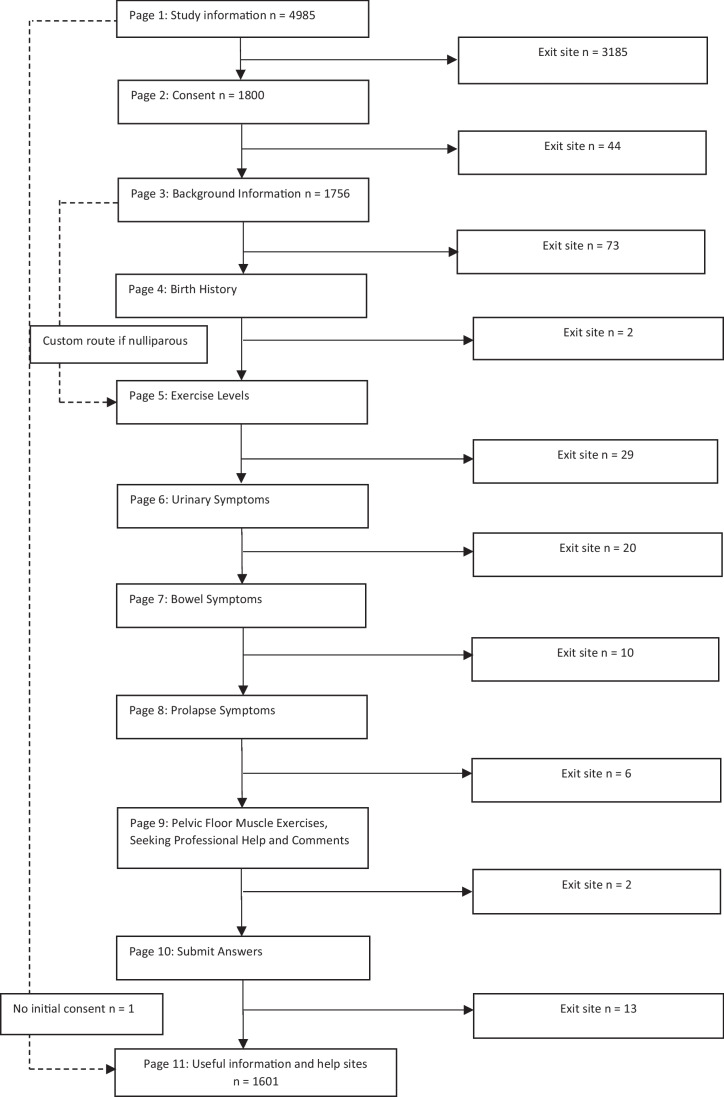
Flow chart to illustrate site visits and points of exit. NB: as the process was anonymous no unique IP addresses were saved so it was not possible to differentiate exits by unique visitors and repeat visits

Visits to the survey site were recorded to be 4,985: 3,185 exiting after the first page (survey information). A few individuals then exited from subsequent pages but most who progressed to consent then completed the survey (Fig. 1). Individual IP addresses were not collected due to the anonymising process so it is not possible to calculate participation rate; each visit recorded could represent duplicate visits by the same participant or unique visits.

In total, 1,600 participants consented to take part and submitted data. Two were excluded: one self-identified as a man, noting that they were male at birth, but wished to underline the need for a similar survey for men; and one did not provide key data regarding birth history, menopausal status and exercise history. Data submitted by the remaining 1,598 participants was analysed: of these 1,141 (71%) reported exercise levels above UK guidelines of more than 150 min per week and 457 (29%) did not exercise or were below this level. Most exercisers (921, 81%) reported doing so for over 5 years and 1,041 (91%) exercised more than three times per week, in line with guidelines. Owing to an initial system issue, 8 participants were able to bypass some questions regarding bowel and vaginal symptoms, which is noted within the results tables.

### Demographics

A majority, 1,359 (85%), of participants were UK based and 144 (9%) were based in the USA, Canada and Australia. All age groups were represented, most, 1,064 (67%), under 50, and 954 (60%) self-identified as being pre-menopausal. The majority, 1,347 (84%), were educated to degree level or above (used to estimate health literacy levels). Average participant BMI was 25.4 kg/m^2^ (SD 5.02, range 15.0–51.6); slightly above normal (18.5–24.9 kg/m^2^. Most, 1,105 (69%), were parous, over half of these reporting two births and 13% experienced a forceps delivery (Table 1).

**Table 1:** Characteristics of participants

Characteristic	Total, *N*=1,598	Non-exercisers, *N*=457 (<2.5 h/week)	Exercisers, *N*=1,141 (>2.5 h/week)
	*n* (%)	*n* (%)	*n* (%)
Age years			
18—36	382 (23.9)	112 (24.5)	270 (23.6)
37–50	682 (42.7)	205 (44.9)	477 (41.8)
51—65	463 (29.0)	112 (24.5)	351 (30.8)
>65	71 (4.4)	28 (6.1)	43 (3.8)
Premenopausal	954 (59.7)	276 (60.4)	678 (59.4)
Menopausal/postmenopausal (on HRT)	147 (9.2)	40 (8.8)	107 (9.4)
Menopausal/postmenopausal (not on HRT)	497 (31.1)	141 (30.9)	356 (31.2)
Education level			
High school	251 (15.7)	74 (16.2)	177 (15.5)
Graduate	584 (36.5)	165 (36.1)	419 (36.7)
Post-graduate	763 (47.8)	218 (47.7)	545 (47.8)
BMI, kg/m^2^, mean (SD, min–max)	25.4 (5.02, 15.0–51.6)	26.7 (5.5, 16.4–33.8)	24.9 (4.75, 15.0–51.6)
Underweight/ normal <24.9 kg/m^2^	925 (57.9)	218 (47.7)	707 (62.0)
Overweight 25–29.9 kg/m^2^	397 (24.8)	120 (26.3)	277 (24.3)
Obese >30 kg/m^2^	276 (17.3)	119 (26.0)	157 (13.7)
Straining to defaecate, *n* (%)	289 (18.1)	99 (21.7)	190 (16.7)
Parity			
0	493 (30.9)	100 (21.9)	393 (34.4)
1	256 (16.0)	86 (18.8)	170 (14.9)
2	616 (38.5)	194 (42.5)	422 (37.0)
3	189 (11.8)	65 (14.2)	124 (10.9)
4+	44 (2.8)	12 (2.6)	32 (2.8)
Forceps	208 (13.0)	68 (14.9)	140 (12.3)

Data presented as frequency (*n*) and % within group; BMI as mean kg/m^2^ (standard deviation, minimum to maximum)

### Main outcomes

#### Prevalence

Bladder: 1,120 (70%, 95% CI 68–72%) of participants reported UI: 592 (37%) SUI, 180 (11%) UUI and 294 (18%) MUI.

Bowel: faecal urgency was reported by 450 (28%, 95% CI 26—30%) participants, 769 (48%, 95% CI 46–51%) reported difficulty controlling flatus and/or stool (AI) and 276 (17%, 95% CI 16—19%) noted marking of underwear by stool.

Prolapse: 293 (18%, 95% CI 17–20%) women noted the sensation of bulging in the vagina.

#### Associations

There were no significant between group differences regarding exercisers and non-exercisers in levels of UI (*p*=0.352), AI (*p*=0.182) or POP (*p*=0.152). Exercisers were less likely to report constipation: 17% compared with 22% of non-exercisers (*p*=0.019; Table 2) .

**Table 2:** Reported symptoms of pelvic floor dysfunction

Pelvic floor disorder	Total group, *N*=1,598	Non-exercisers <2.5 h/week, *N*=457	Exercisers >2.5 h/week, *N*=1,141	Between-group difference
	*n* (%)	*n* (%)	*p* (%)	
Urinary incontinence	1,120 (70.1)	328 (71.8)	792 (69.4)	*p*=0.352
UI severity (ICIQ scale)^a^				*p*=0.406^b^
Slight	423 (26.5)	119 (26.0)	304 (26.6)	
Moderate	506 (31.7)	140 (30.6)	366 (32.1)	
Severe	191 (12.0)	66 (14.4)	125 (11.0)	
very severe	12 (0.8)	4 (0.9)	8 (0.7)	
Anal incontinence (gas/stool)	769 (48.1), missing (1)	208 (45.5)	561 (49.2), missing (1)	*p*=0.182
Anal incontinence (marking underwear)	278 (17.3), missing (1)	78 (17.1)	198 (17.4), missing (1)	*p*=0.886
Faecal urgency	450 (28.2)	132 (28.9)	318 (27.9)	*p*=0.684
Constipation	289 (18.1)	99 (21.7)	190 (16.7)	*p*=0.019*
Pelvic organ prolapse	293 (18.4)	94 (20.6), missing (1)	199 (17.4)	*p*=0.152

All presented as frequency and within-group percentage

*UI* urinary incontinence, *ICIQ* International Consultation Incontinence Questionnaire

*Reached between-group significance, Pearson Chi-squared test (*p*<0.05)

^a^ICIQ-UI-SF severity index based on Klovning et al. [20]: maximum score = 21; slight (1–5), moderate (6–12), severe (13–18) and very severe (19–21)

^b^All reported with 1 degree of freedom other than ICIQ severity score with 4 degrees of freedom and one overall *p* value

After regression analysis using logistic binomial regression to account for other risk factors (age, reduced oestrogen, BMI, constipation, parity and forceps delivery) with non-exercisers (<2.5 h/week) as the reference group, no significant association was found between recreational exercise and PFD.

Risk factors associated with UI included aging, BMI, constipation and parity. AI was associated with age, constipation and forceps delivery. POP was associated with hormonal status, constipation and increasing parity (Table 3).

**Table 3:** Adjusted odds ratios for the relationship between pelvic floor symptoms and risk factors

Risk factors	Adjusted OR (95% CI)	*p* value	Adjusted OR (95% CI)	*p* value	Adjusted OR (95% CI)	*p* value
	UI		POP		AI	
Exercise >2.5 h/week	1.092	0.504	1.00 (0.75–1.35)	0.972	1.21 (0.96–1.52)	0.105
Age (years)						
37–50	1.97 (1.47–2.63)	<0.001*	1.20 (0.80–1.80)	0.379	1.82 (1.04–3.19)	0.038*
51—65	2.10 (1.38–3.18)	<0.001*	1.16 (0.69–1.94)	0.585	1.83 (0.92–3.64)	0.087
>65	2.54 (1.23–5.27)	0.012*	1.03 (0.45–0.99)	0.936	3.28 (1.34–8.08)	0.010*
Hormonal effects	0.99 (0.69–1.42)	0.965	0.66 (0.45–0.99)	0.044*	1.49 (0.92–2.41)	0.109
BMI kg/m^2^	1.07 (1.04–1.10)	<0.001*	1.00 (0.97–1.02)	0.723	1.02 (1.00–1.04)	0.109
Constipation	1.40 (1.03–1.90)	0.032*	1.98 (1.44–2.73)	<0.001*	1.32 (1.02–1.72)	0.037*
Parity						
1	1.71 (1.21–2.42)	0.003*	5.45 (3.14–9.46)	<0.001*	0.79 (0.57–1.10)	0.159
2	1.89 (1.41–2.52)	<0.001*	7.33 (4.43–12.13)	<0.001*	0.87 (0.67–1.14)	0.317
3	2.32 (1.50–3.59)	<0.001*	8.94 (5.01–15.95)	<0.001*	0.93 (0.64–1.35)	0.697
4+	1.95 (0.92–4.12)	0.081	14.02 (6.43–30.57)	<0.001*	1.30 (0.68–2.49)	0.430
Forceps	1.16 (0.80–1.68)	0.443	1.27 (0.90–1.79)	0.182	1.58 (1.16–2.16)	0.004*

Calculated via binomial logistic regression analysis

Risk factors: age (years) (18–36 years group as reference); hormonal effects (premenopausal group as reference); BMI; constipation (no straining to defaecate as reference); parity (nulliparous as reference); forceps delivery (non-forceps delivery as reference), exercise group (exercise levels <2.5 h/week as reference group)

*BMI* body mass index, *OR* odds ratio, *CI* confidence interval, *UI* urinary incontinence, *POP* pelvic organ prolapse, *AI* anal incontinence

*Reached significance in regression analysis. Adjusted for all risk factors noted

Subdivision of exercise levels based on hours/week: (2.5–6 h, 6–10 h and > 10 h) showed no significant differences regarding prevalence of UI. Women who exercised >10 h/week reported fewer incidences of POP (OR 0.70, CI 95% 0.42–1.19), but this was not significant (*p*=0.190). There was, however, increased reporting of AI by those exercising >10 h/week: adjusted OR of 1.48 (CI 95%, 1.04–2.10 (Table 4).

**Table 4:** Adjusted odds ratios to indicate any relationship between levels of exercise and pelvic floor symptoms

Subdivided by exercise levels	Adjusted OR (95% CI)	*p* value	Adjusted OR (95% CI)	*p* value	Adjusted OR (95% CI)	*p* value
	UI		POP		AI	
2.5–6 h/week	1.09 (0.82–1.44)	0.550	1.06 (0.77–1.46)	0.716	1.14 (0.89–1.47)	0.291
6–10 h/week	1.23 (0.87–1.74)	0.238	1.06 (0.70–1.58)	0.790	1.22 (0.90–1.65)	0.211
>10 h/week	0.92 (0.63–1.35)	0.660	0.70 (0.42–1.19)	0.190	1.48 (1.04–2.10)	0.031*

Non-exercisers as the reference group and adjusted for all risk factors as in Table 3

*OR* odds ratio, *CI* confidence interval, *UI* urinary incontinence, *POP* pelvic organ prolapse, *AI* anal incontinence

*Reached significance in regression analysis

Further investigation to account for potential effects of exercise involving impact only as opposed to non-impact sport revealed no significant differences in levels of PFD (Table 5).

**Table 5:** Adjusted odds ratios for the relationship between types of exercise and pelvic floor symptoms

Subdivided by exercise type	Adjusted OR (95% CI)	*p* value	Adjusted OR (95% CI)	*p* value	Adjusted OR (95% CI)	*p* value
	UI		POP		AI	
Both: low and high-impact	0.78 (0.57–1.09)	0.143	0.73 (0.49–1.09)	0.122	1.18 (0.88–1.59)	0.268
High-impact only	0.84 (0.59–1.20)	0.342	0.92 (0.60–1.42)	0.715	0.86 (0.62–1.19)	0.374

Low-impact group as reference group for relationships

*OR* odds ratio, *CI* confidence interval, *UI* urinary incontinence, *POP* pelvic organ prolapse, *AI* anal incontinence

#### Pelvic floor exercises and treatments

Pelvic floor exercises were performed regularly by only 646 (40%) participants.

Of those reporting any pelvic floor dysfunction, only 450 out of 1,319 (34%) had sought professional help. Those with symptoms were no more or less likely to exercise their pelvic floors.

#### Responses to open question

In the final section, 537 participants made comments. These are reported in detail elsewhere [22] but we report, in brief, key illustrative quotations regarding impact of symptoms on access to sport and treatments to manage symptoms.

#### Pelvic floor symptoms impacting access to sport

Often participants commented that their pelvic floor symptoms were the reason why they could no longer take part in sport and exercise:

“I would love to exercise to lose weight, but it is impossible with these bladder issues … it's so frustrating”

“…my exercise intensity and frequency have changed since having children due to leakage/prolapse symptoms. Before kids my exercise intensity was high and 6 days/week. Now, I don’t engage in high intensity exercise anymore….”

Many commented on the negative effects of this, ranging from ensuring that their bladder had been emptied before leaving the house:

“I feel like I should always empty my bladder before I leave the house, gym, work etc. to avoid a panic when I need to urinate.”

to great distress:

“It's impacted my life; I can't run anymore …. No one gives a damn because it's only women.”

#### Treatment

Some commented on treatments they had attempted to seek or been offered, and many suggested that pelvic floor muscle exercises did help:

“Doing daily regular sustained pelvic floor exercises has greatly improved my symptoms”

whereas others found that taking part in sport had helped:

“I started to include weight training …. and feel that has helped my pelvic floor enormously.”

## Discussion

The objectives of this survey were to determine the levels of PFD in women who exercised at or above recommended guidelines for healthy living and in those who did not and to identify any correlation between exercising recreationally and the incidence of PFD, as previously noted in literature regarding elite athletes [11, 12, 23, 24].

All levels of PFD reported were high compared with other studies: UI was reported by 40% of women in a 2015 UK survey [16] compared with 70% of our participants, and AI was reported by only 14% of women in a US epidemiological survey [25] compared with 48% in this survey. However, a recent study investigating the long-term effects of sphincter injuries at birth on AI reported 60% prevalence of AI in the control group (those without sphincter injury) [26] and this level was similar to that found in a group of young, nulliparous women [12]. Levels of POP again appear to be greater than that reported in the US epidemiological study [25] but in another recent internet-based survey, 14% of participants reported POP [13] compared with 18% of our respondents. It is likely that there will be some selection bias in an internet survey as women with an interest are more likely to take part, despite advertisements aiming to recruit ALL women. However, it may be that as pelvic health symptoms are increasingly being discussed more openly in the media women are becoming more confident to share information regarding these symptoms.

We found no significant associations between taking part in recreational sport and exercise and PFD other than a small increase in the number of women reporting AI when exercising for more than 10 h/week. However, this should be interpreted with caution given the low numbers of women exercising at higher levels in this survey. It is important to note that many previous studies reporting increased levels of PFD in athletes have investigated the elite population [11], whereas other investigations that have noted significantly higher levels of UI included only young nulliparous women, without the increased extra risks associated with parity and/or assisted delivery in their sedentary cohort [12]. Athletes in the latter study reported training on average for 19 h/week, whereas the majority of our exercisers were exercising for less than 10 h/week and a positive association has been reported between volume of physical activity and the frequency of UI [27]. However, a previous study also found no significant correlation between UI or POP and exercise, and the only significant correlation reported was between AI and sport [28].

The demands of elite level competition dictate that reducing training levels or modifying load is rarely an option unless there is illness or injury. It is therefore likely that elite athletes, many of whom have never mentioned their symptoms to anyone [29], would not alter their sport or training levels as a result of PFD. In the case of the recreational athletes in our survey, however, comments suggested that women often modified their sports to include lower impact activities or reduced the level of exercise they took part in altogether. This, combined with the lower volume of exercise performed by most of our exercisers, may explain the differences in the results. However, it should also be noted that as the majority of exercisers in our survey have been doing so for over 5 years there is little in this study to suggest that recreational sport at these levels is a specific risk to the pelvic floor.

Finally, the majority of those who reported PFD here had not sought professional help, despite comments suggesting that PFD caused distress. This is recognised and has previously been reported in other studies on both athletes [29] and the general population [16].

A major strength of internet-based surveys is the ability to recruit large numbers of participants from a spread of geographical locations. Moreover, although self-reporting of symptoms may be less accurate than using objective measures such as pad tests to detect incontinence or vaginal examination to diagnose POP, this is mitigated by the use of validated questionnaires to predict symptoms.

There are, however, associated limitations, not least of which is the possibility of selection bias, as those affected by the criteria being investigated are most likely to take part, which may increase prevalence levels. In addition, although advertisements asked ALL women to participate, the only inclusion criteria were to be adult and female; this could mean that some were pregnant or possessed disabilities that could have an impact on their pelvic floor function. Further, although we aimed to recruit a diverse population, the majority of participants were educated to degree level or above. It is then even more surprising that most symptomatic participants had never sought professional help.

## Conclusion

Overall levels of PFD within this survey are high but there was no association between recreational exercise and the rates of PFD reported. Further longitudinal studies may help to investigate any long-term risks of recreational exercise to pelvic health. However, based on the results of this survey and the multiple health benefits associated with taking part in regular sport and exercise, women and health professionals should be cautious when extrapolating the risks to the pelvic floor associated with elite sport to recreational exercisers.

### Supplementary information

Below is the link to the electronic supplementary material.

Supplementary file 1 (DOCX 23 KB)

Supplementary file 2 (DOCX 33 KB)

## Declarations

### Data sharing

A summary of the data set generated and analysed during the current study may be available from the corresponding author on reasonable request.

### Public and patient involvement

A patient representative, who has experienced pelvic floor dysfunction and its impact on her sport, was involved as a member of the study steering group from its inception and took an equal part in all aspects of design of the survey, study protocol and decisions regarding advertising and analysis. She will continue to be involved as a member of the steering group to decide on potential ways to disseminate the results to the public going forward. Further, local women were recruited to pilot initial versions of the survey to identify any issues with wording and content. The final version was modified based on their feedback.
