# Supplementary material for: Prevalence of pelvic floor dysfunction in recreational athletes: a cross-sectional survey
Source: Int Urogynecol J. 2023 May 10;34(10):2429–37. doi: 10.1007/s00192-023-05548-8 (PMC10590299; doi:10.1007/s00192-023-05548-8)
Supplement: Supplementary file 1 — (DOCX 23 KB) [file 192_2023_5548_MOESM1_ESM.docx]

**Supplemental Table 1. Checklist for Reporting Results of Internet E-Surveys (CHERRIES)**

| ***Checklist Item*** | ***Explanation*** | ***Page Number*** |
| --- | --- | --- |
| Describe survey design | Target population: convenience sample of adult women, including those who experienced pelvic floor disorders and those who did not, including those who exercise for over 150’/week and those who do not | Page 4 |
| IRB approval | Faculty of Medicine & Health Sciences Research Ethics Committee (Reference FMHS 501-0322) | Page 15 |
| Informed consent | Participants were informed of the nature of the investigation (to explore any differences in the prevalence of pelvic floor disorders in women who exercise regularly compared with those who do not). They were informed of the likely time to complete the survey (approximately 10 minutes, calculated from the average time to complete in a pilot of the survey). They were informed that all data would be collected anonymously and that there was no link to any IP address. They were given the email address of the principal investigator, should they require any further information and also the name and email address of the Head of the School of Health Sciences, should they wish to complain). They were informed that all data would be stored on the University of Nottingham server in encrypted files. Participation was completely voluntary with options for those that wished to close the window and withdraw at any point. Those that refused consent were taken to the last page of the survey which only contained links to help and advice sites such as the appropriate page of the NHS website, the POGP website (specialist UK pelvic health physiotherapy website) and the Bladder and bowel foundation, no data was then collected. | Not reported in article |
| Data protection | Email addresses were collected only from those wishing to be contacted for further studies or to clarify results in a separate survey, there was no link from these addresses to the survey data collected. No other personal identifying information was collected. The survey was hosted by Jisc (formerly BOS) and all data stored on a secure server. | Not included in the article |
| Development and testing | Survey development was supported by all investigators and the steering group, which included a specialist pelvic health physiotherapist, a professor of rehabilitation research, a professor of sports medicine and an PPI member. The online survey was piloted with colleagues from the School of Health Science and an external clinical practice. Both groups included professionals and admin staff. | Page 4 |
| Open survey versus closed survey | This was an open survey with no requirement for password control. | Not included in the article |
| Contact mode | Advertisements were posted via Social Media networks: Facebook, LinkedIn, Twitter, Instagram and via snowball methods. Some posters were distributed via physiotherapy clinics and their websites with QR codes linking to the survey. Posters were distributed to sports clubs for sharing with QR codes to link to the survey. | Page 4 |
| Advertising the survey | Banner advertisements were shared on social media sites as above. Announcement: ‘Are you female and over 18? Help us answer important questions about pelvic floor issues in women who exercise.  We need answers from women who exercise and those who do not YOU could help us answer important  questions about this topic in just 10 minutes.’ | Not included in the article |
| Web/E-mail | The survey was distributed via websites and social media platforms as listed above. | Page 4 |
| Context | Initial sharing was via investigators Facebook pages and the Facebook page of the UK specialist pelvic health website, two physiotherapy websites and then shared from these sites to the wider Facebook community. It was shared via LinkedIn via the PI’s business account, the Rowing British Professional Women’s Network, UK Diving Network. Further it was shared via the This Girl Can Strava chat function. Shared on Twitter via the investigators Twitter accounts and then more widely from there. Local runners shared the post and posters to their running groups. It was shared on the Marston and Carlsberg menopause Facebook site and then more widely throughout their European Facebook site by their HR department. It was shared via women’s WhatsApp groups for a London rowing club. These are to name the contexts that are known by the investigators, but onward sharing was beyond their influence. | Not in detail in article |
| Mandatory/voluntary | Participation was completely voluntary with options for those that wished to close the window and withdraw at any point, no data was collected until the participant chose to submit their answers at the end. | Not in article |
| Incentives | None | Not in article |
| Time/Date | The survey was available from 6^th^ May till the 31^st of^ July 2022. | Page 5 |
| Randomization of items or questionnaires | There was no randomisation of the survey questions rather sections were built to allow for adaptive questioning. | Not in article |
| Adaptive questioning | Questions pertaining to each subject area were divided into pages in order that participants could bypass some questions that did not apply to them. That is nulliparous participants did not require to answer questions about childbirth. | Not in article |
| Number of Items | The survey contained 37 total possible questions (including the consent and submit pages) which took on average 10 minutes to complete. There were 9 pages in total including the final submission page and on average there were 4 questions per page, range 1 to 8. | Not in article |
| Number of screens (pages) | The total number of pages possible was 9, including consent and submit pages. | Not in article |
| Completeness check | All question items provided a non-response option (either ‘other’ with an option to add specific data or not applicable). All questions required a response to advance to subsequent question screens, apart from one open comment question at the end which allowed participants to comment in their own words regarding any of the issues that had been raised, this was not compulsory. A completeness check was conducted after the questionnaire was submitted during the analysis phase. | Page 5,6 |
| Review step | Participants were not required to review their responses; however a “Prev” button was provided if participants wished to edit previous answers. | Not in article |
| Unique site visitor | IP addresses were not able to be tracked as the software Jisc provides does not allow for this in order that anonymity is maintained. | Page 6 |
| View rate (Ratio of unique survey visitors/unique site visitors) | Not able to calculate as the anonymity of the survey does not allow IP addresses to be viewed. From a total of 4985 site visits 1800 participants, progressed to page 2 (consent) one did not consent so progressed directly to the ‘Finish’. Others exited the site at various points and 1600 progressed to the final’ Submit’ stage. | Page 6 and see Figure 1 |
| Participation rate (Ratio of unique visitors who agreed to participate/unique first survey page visitors) | Not able to calculate as not know if all site visits were unique. | Not in article |
| Completion rate (Ratio of users who finished the survey/users who agreed to participate) | The survey did not allow participants to submit partially completed an incomplete survey, so this is not possible to calculate. Only one did not consent but many others may have left the survey without completing. | See Figure 1 |
| Cookies used | No cookies used to preserve anonymity. | Not in article |
| IP check | IP addresses were not collected from participants to ensure anonymity. | Page 6 |
| Log file analysis | Log file analysis was not utilized. | Not in article |
| Registration | Not applicable |  |
| Handling of incomplete questionnaires | Not applicable. |  |
| Questionnaires submitted with an atypical timestamp | Not used. |  |
| Statistical correction | Logistic binomial regression analysis used to adjust for risk factors | Page 6 |

This checklist has been modified from Eysenbach G. Improving the quality of Web surveys: the Checklist for Reporting Results of Internet E-Surveys (CHERRIES). J Med Internet Res. 2004 Sep 29;6(3):e34 [erratum in J Med Internet Res. 2012; 14(1): e8.]. Article available at [https://www.jmir.org/2004/3/e34](https://www.jmir.org/2004/3/e34/)/; erratum available <https://www.jmir.org/2012/1/e8/>. Copyright ©Gunther Eysenbach. Originally published in the [Journal of Medical Internet](http://www.jmir.org) Research, 29.9.2004 and 04.01.2012.
